# Opioid use following cardio-thoracic intensive care: risk factors and outcomes: a cohort study

**DOI:** 10.1038/s41598-023-50508-3

**Published:** 2024-01-02

**Authors:** Erik von Oelreich, Francesca Campoccia Jalde, Susanne Rysz, Jesper Eriksson

**Affiliations:** 1 Perioperative Medicine and Intensive Care, Karolinska University Hospital, Solna, Stockholm, Sweden; 2 Section of Anesthesiology and Intensive Care Medicine, Department of Physiology and Pharmacology, Karolinska Institutet, 171 65 Solna, Stockholm, Sweden

**Keywords:** Epidemiology, Outcomes research

## Abstract

Opioid misuse has become a serious public health problem. Patients admitted to cardio-thoracic critical care are usually exposed to opioids, but the incidence and effects of chronic opioid use are not known. The primary objective was to describe opioid use after admission to a cardio-thoracic intensive care unit. Secondary objectives were to identify factors associated with chronic opioid usage and analyze risk of death. This cohort study included all cardio-thoracic ICU care episodes in Sweden between 2010 and 2018. Among the 34,200 patients included in the final study cohort, 4050 developed persistent opioid use after ICU care. Younger age, preadmission opioid use, female sex, presence of comorbidities and earlier year of ICU admission were all found to be associated with persistent opioid use. The adjusted hazard ratio for mortality between 6 and 18 months after admission among individuals with persistent opioid use was 2.2 (95% CI 1.8–2.6; *P* < 0.001). For opioid-naïve patients before ICU admission, new onset of chronic opioid usage was significant during the follow-up period of 24 months. Despite the absence of conclusive evidence supporting extended opioid treatment, the average opioid consumption remains notably elevated twelve months subsequent to cardio-thoracic ICU care.

## Introduction

Opioids are powerful analgesics and are therefore considered a primary treatment for moderate to severe pain^1^. However, opioid use carries risks such as addiction and physical dependence^2^ and prolonged use can result in increased dosage due to tolerance^3^. The misapplication of prescription opioids constitutes a notable worldwide health concern and contributes to the global disease burden^4^. As opioid use continues to rise, the treatment of acute and chronic pain has become an increasingly challenging issue in modern medicine^5^.

Prolonged administration of opioids is commonly defined as treatment extending beyond three months^6^. Several determinants have been linked to long-term opioid, including the amount prescribed in the initial period after injury^7^, level of inpatient use^8^, and pre-injury opioid use^9^. Additional potential factors include comorbid mental health disorders and a history of illicit drug and alcohol abuse^10,11^. Prescription of opioids seem to exhibit a pattern where individuals with a high likelihood of adverse outcomes after treatment with opioids, are also more prone to be prescribed substantial volumes of opioids.

The existing body of evidence concerning extended, persistent usage is limited, while conversely, there exists a substantial body of evidence pointing to adverse outcomes in cases outside of short-term therapeutic regimens. Absence of randomized controlled trials investigating the efficacy of opioids is notable. Furthermore, despite their widespread utilization to treat severe pain, opioids are still frequently misused. Most of the current research on opioids is conducted in the United States, where the majority of prescribed opioids are consumed^12^. In the United States, over 30% of adults use prescription opioids, and in 2019, over 70,000 drug overdose deaths occurred^13^. Currently, prescription opioids and heroin are the primary contributors to drug overdose deaths^14^, primarily originating from opioids prescribed within recommended guidelines^15^.

Furthermore, several patients suffer from prolonged pain after ICU discharge, and despite the absence of evidenced benefits from persistent opioid usage, opioids are frequently employed in this context^16^. Due to the escalating prescription of opioids, older studies might not accurately reflect contemporary prescription trends, leading to challenges in interpreting findings. A contemporary ICU study from Canada could show that merely 2.6% of mechanically ventilated patients, fulfilled the criteria for persistent opioid use post-hospital discharge^17^.

The administration of opioids for pain management or sedation is common among ICU patients. The extent of persistent opioid utilization after critical care is not completely understood, but theories that prolonged intravenous opioid infusions during ICU care may contribute to persistent use after discharge has not found support in previous studies^18,19^. One previous study showed that thoracic surgical patients are more likely than other surgical patients to develop chronic opioid use after surgery^20^. However, another study on patients undergoing thoracic surgery did not identify any procedural factors that contributed to new persistent opioid use and the relevance of persistent opioid use following cardio-thoracic critical care remains unknown.

The primary aim was to describe opioid usage following admission to cardio-thoracic critical care. Secondary aims included determining risk factors for persistent opioid usage after critical care admission and examining whether persistent opioid usage after critical care is associated with an elevated risk of mortality.

## Methods

### Ethics approval

The Regional Ethical Review Board in Stockholm, Sweden, approved the study (approval numbers 2018/2541-31 and 2019–00,213) and waived requirement for informed consent. All research was performed in accordance with national guidelines and regulations.

### Study design

A cohort was established using data from the Swedish Intensive Care Registry (SIR) between 2010 and 2018. SIR is a national quality register for intensive care and collects information from ICUs in Sweden including data on demography, procedures, and mortality^21^. If patients had more than one care episode registered, the first one was included. Data on comorbidities was collected from the Swedish National Patient Register^22^ up to five years before ICU admission. Socioeconomic factors including education were collected from the Longitudinal Integration Database for Health Insurance and Labour Market Studies (LISA)^23^. Education level at the time of ICU admission was defined as low, medium, or high, corresponding to 9 years or less (primary school), 10–12 years (secondary school) and more than 12 years (university level), respectively. Income in the year before admission was classified into low, medium, and high corresponding to less than half of the median national income, between half to double the median national income and more than double the median national income, respectively. Data on mortality and prescribed drugs was assessed using The Swedish Cause of Death Register^24^ and The Swedish Prescribed Drug Register^25^.

### Outcomes

Primary aim was persistent opioid usage after critical care admission and the secondary aim was all-cause mortality 6–18 months after admission to critical care.

### Definitions

Use of opioids before ICU admission equaled at least one written and dispensed prescription within the 12 months preceding ICU admission. Persistent opioid use was defined as a minimum of two prescriptions within the first six months (180 days) following ICU admission^6^. Equipotent doses were computed deploying Oral Morphine Equivalents (OMEQ) to compare opioids with differing potency (Table S1 in the Supplementary Information provides a list of the opioids included along with their corresponding conversion rates). Individuals who died during the first quarter (three months) after ICU admission were excluded from the analysis. Patients who had not used opioids within one year before ICU admission were analyzed separately and referenced as opioid naïve. Individuals with an established use of methadone and/or specific preparations of buprenorphine (coded under Anatomical Therapeutic Chemical code (ATC) code N07BC), primarily utilized as opioid agonist treatment for patients with substance use disorders, were excluded from the analysis.

### Statistical analysis

Generalized Estimating Equations (GEE) regression models were performed when exploring differences in mean opioid use pre and post admission to critical care. Cox proportional hazard models were employed to examine the relationship between persistent opioid use and all-cause mortality within the timeframe of 6–18 months post-ICU admission. Known or potential confounders were selected before the study. Multivariable logistic regression models were used when estimating odds ratios (OR) for associations between persistent opioid use and clinically relevant risk factors.

### Sensitivity analysis

To assess non-random dropout due to death, probability weights were used in a multivariable logistic regression model^26^. The probability of dying within the first 3 months following admission to critical care was estimated in a logistic regression model which included all covariates in the multivariable model.

*P* value < 0.05 was considered statistically significant; all tests were two-tailed. Data were analyzed using Stata/SE 16.1. The study adhered to the Strengthening the Reporting of Observational Studies in Epidemiology (STROBE) recommendations for cohort studies^27^.

## Results

**Figure 1:**
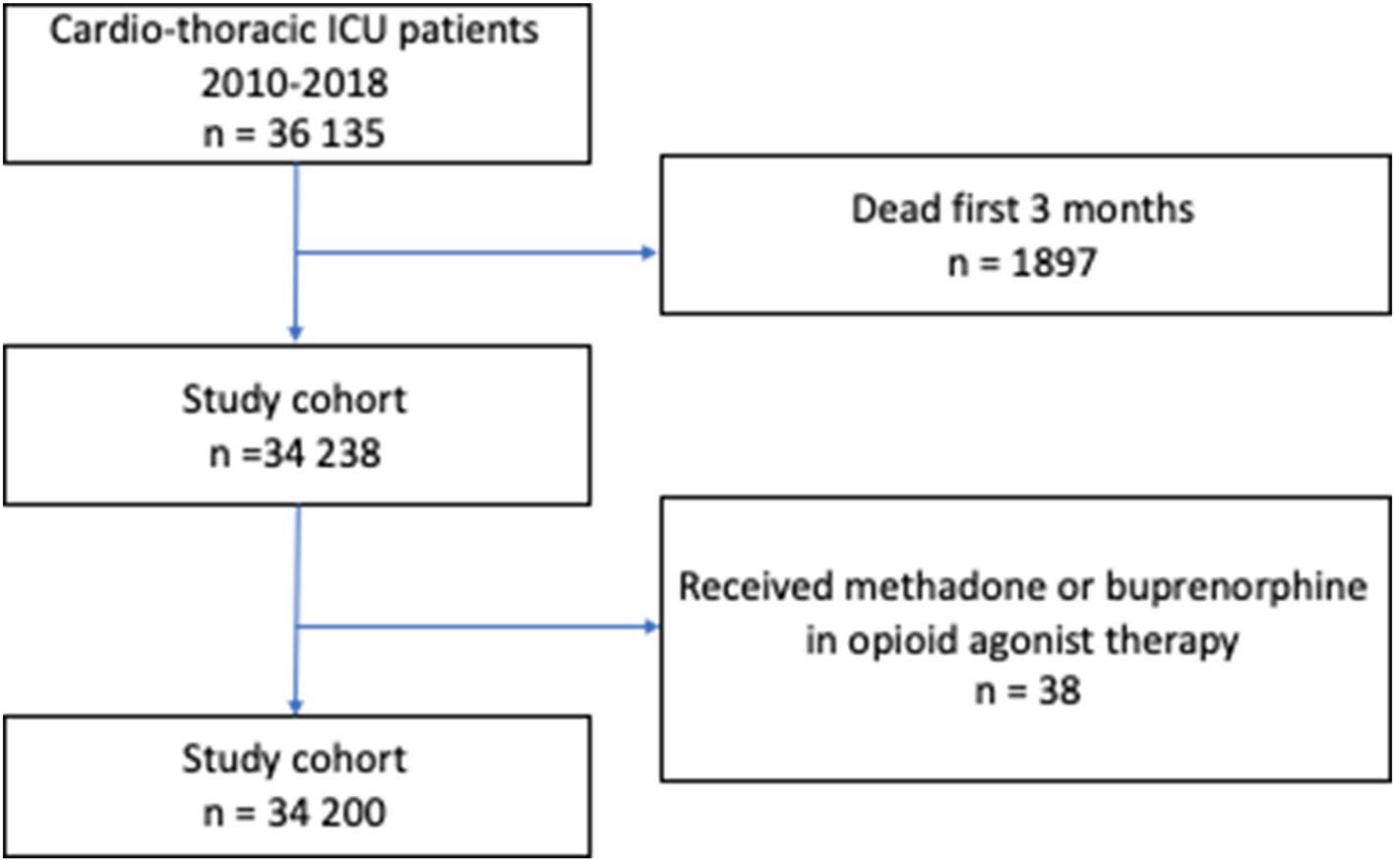
Flow chart of included patients.

**Figure 2:**
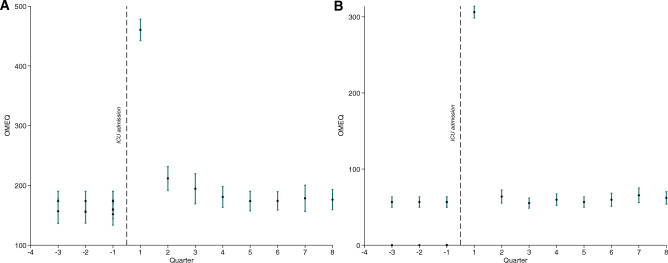
Opioid prescription in relation to ICU care. Opioid prescription pre- and post-ICU care for the entire study cohort (n = 34,200) (**A**) and a subset of patients not using opioids 12 mo prior to ICU admission (n = 29,390) (**B**). OMEQ = oral morphine equivalents.

Between 2010 and 2018, altogether 36,135 patients were included in SIR as cardio-thoracic ICU patients. After excluding 1897 individuals dying in the first quarter period after admission to the ICU and 38 individuals receiving methadone or buprenorphine, 34,200 patients made up the final study cohort (Fig. 1). The final study cohort is presented in Table 1. Opioid use (with 95% confidence intervals (CI)) for the final cohort (n = 34,200) and for a subset of opioid naïve individuals (not using any opioids during a 12 month-period preceding ICU admission, n = 29,390) are presented in Fig. 2a,b.

**Table 1:** General characteristics for patients included.

ICU patients	
Count	34 200
Age categories	
18–45	2076 (6.1)
46–60	6820 (19.9)
61–70	11,500 (33.6)
71–80	11,290 (33.0)
80–	2514 (7.4)
Male, count (%)	24 800 (72.5)
Income categories, count (%)	
Low	3423 (10.0)
Medium	27 837 (81.7)
High	2819 (8.3)
Education level, count (%)	
Low	10 803 (32.1)
Medium	15 580 (43.3)
High	8303 (24.6)
CCI categories, count (%)	
CCI 0	9748 (28.5)
CCI 1	10 799 (31.6)
CCI > 1	13 653 (39.9)
Psychiatric comorbidity, count (%)	2044 (6.0)
Substance abuse, count (%)	729 (2.1)
Opioid use 6 months pre-ICU	4810 (14.1)
Acute myocardial infarction	10 169 (29.7)
Congestive heart failure	6567 (19.2)
Peripheral vascular disease	5157 (15.1)
Cerebrovascular disease	2946 (8.6)
Dementia	70 (0.2)
COPD	3739 (10.9)
Rheumatoid disease	1190 (3.5)
Peptic ulcer disease	604 (1.8)
Mild liver disease	482 (1.4)
Moderate/severe liver disease	107 (0.3)
Diabetes w/o complications	7186 (21.0)
Diabetes with complications	2036 (6.0)
Hemiplegia or paraplegia	235 (0.7)
Renal disease	1465 (4.3)
Cancer	3235 (9.5)
Metastatic cancer	310 (0.9)
AIDS	38 (0.1)
ICU length of stay, days	
0–2	24 937 (72.9)
3–7	7460 (21.8)
> 7	1803 (5.3)
Surgery	
Acute care	2201 (6.4)
Elective	30 179 (88.2)
No surgery	1820 (5.3)
ICU admission year	
2010–2011	7706 (22.5)
2012–2013	7433 (21.7)
2014–2015	7154 (20.9)
2016–2018	11 907 (34.8)

Categorical parameters are presented as n (%), continuous parameters as median with interquartile range (IQR), *CCI* Charlson Comorbidity Index, *COPD* chronic obstructive pulmonary disease, *AIDS* acquired immune deficiency syndrome, *ICU* intensive care unit.

Mean opioid use increased in the quarter period before admission to critical care. After admission, there was a peak in the first three-month period followed by a decline during the first year. After 12 months, the mean consumption returned to baseline levels (equaling 9–12 months before admission), as shown in the Supplementary Information, Table S2. Characteristics for the patients with and without prior opioid consumption are presented in the Supplementary Information, Table S3. Among opioid-naïve patients, the mean opioid consumption remained unchanged between 3 months after admission up to 24 months of follow up.

Cardiac patients admitted to critical care who subsequently developed persistent opioid usage (n = 4050) were younger, less likely to be male, had lower level of education and income, and had more psychiatric and somatic disorders as well as more substance dependence at the time of admission to critical care. In addition, more patients had emergency surgery, length of stay in the ICU was longer, 40% of persistent opioid users also used opioids before admission to critical care (Table 2). Also among patients not using opioids prior to critical care, similar differences were seen between patients with and without persistent opioid use (Supplementary Information, Table S4).

**Table 2:** General characteristics in included ICU patients stratified by chronic opioid use during the first six months after ICU care.

	No chronic opioid use	Chronic opioid use
Count	30 150	4050
Age categories		
18–45	1780 (5.0)	296 (7.3)
46–60	5735 (19.0)	1085 (26.8)
61–70	10 115 (33.5)	1385 (34.2)
71–80	12 209 (33.9)	1081 (26.7)
80-	2311 (7.7)	203 (5.0)
Male, count (%)	22 180 (73.5)	2620 (64.7)
Income categories, count (%)		
Low	2943 (9.8)	480 (11.9)
Medium	24 549 (81.7)	3288 (81.6)
High	2557 (8.5)	262 (6.5)
Education level, count (%)		
Low	9539 (32.1)	1264 (31.8)
Medium	12 696 (42.7)	1884 (47.4)
High	7480 (25.2)	823 (20.7)
CCI categories, count (%)		
CCI 0	8870 (29.4)	878 (21.7)
CCI 1	9658 (32.0)	1141 (28.2)
CCI > 1	11 622 (38.5)	2031 (50.1)
Psychiatric comorbidity, count (%)	1584 (5.3)	460 (11.4)
Substance abuse, count (%)	532 (1.8)	197 (4.9)
Opioid use 6 months pre-ICU	3023 (10.0)	1787 (44.1)
Acute myocardial infarction	8840 (29.3)	1329 (32.8)
Congestive heart failure	5708 (18.9)	859 (21.2)
Peripheral vascular disease	4423 (14.7)	734 (18.1)
Cerebrovascular disease	2575 (8.5)	371 (9.2)
Dementia	59 (0.2)	11 (0.3)
COPD	3004 (10.0)	735 (18.1)
Rheumatoid disease	983 (3.3)	207 (5.1)
Peptic ulcer disease	488 (1.6)	116 (2.9)
Mild liver disease	353 (1.2)	129 (3.2)
Moderate/severe liver disease	92 (0.3)	15 (0.4)
Diabetes w/o complications	6125 (20.3)	1061 (26.2)
Diabetes with complications	1678 (5.6)	358 (8.8)
Hemiplegia or paraplegia	199 (0.7)	36 (0.9)
Renal disease	1253 (4.2)	212 (5.2)
Cancer	2746 (9.1)	489 (12.1)
Metastatic cancer	238 (0.8)	72 (1.8)
AIDS	29 (0.1)	9 (0.2)
ICU length of stay, days		
0–2	22 146 (73.5)	2791 (68.9)
3–7	6427 (21.3)	1033 (25.5)
> 7	1577 (5.2)	226 (5.6)
Surgery		
Acute care	1891 (6.3)	310 (7.7)
Elective	26 692 (88.5)	3487 (86.1)
No surgery	1567 (5.2)	253 (6.2)
ICU admission year		
2010–2011	6684 (22.2)	1022 (25.2)
2012–2013	6564 (21.8)	869 (21.5)
2014–2015	6294 (20.9)	860 (21.2)
2016–2018	10 608 (35.2)	1299 (32.1)

Categorical parameters are presented as n (%), continuous parameters as median with interquartile range (IQR), *CCI* Charlson comorbidity index, *COPD* chronic obstructive pulmonary disease, *AIDS* acquired immune deficiency syndrome, *ICU* intensive care unit.

In the multivariable logistic regression analysis, determinants associated with higher odds of chronic opioid use included female sex, psychiatric and somatic comorbid conditions, substance dependence, preadmission opioid usage, and critical care stay for 3–7 days. In contrast, high age, high income and education, ICU stay for more than 7 days, and admission year 2016–2018 were all associated with lower odds of persistent opioid usage (Table 3). Patients not using opioids prior to critical care, determinants associated with increased odds of chronic opioid use included female sex, medium level of education, psychiatric and somatic comorbidities, substance abuse, and length of stay in the ICU for 3–7 days. Higher age and admission year 2012–2013 or 2016–2018 were associated with lower odds of chronic opioid use (Supplementary Information, Table S5).

**Table 3:** Univariate and multivariable logistic regression analyses, associations with chronic opioid use presented as OR (95% CI).

	Univariate	*P* value	Multivariable	*P* value
*Age categories*				
18–45	Ref.	Ref.	Ref.	Ref.
46–60	1.14 (0.99–1.31)	0.069	1.05 (0.90–1.22)	0.54
61–70	0.82 (0.72–0.94)	0.005	0.73 (0.63–0.85)	< 0.001
71–80	0.64 (0.55–0.73)	< 0.001	0.53 (0.45–0.62)	< 0.001
80-	0.53 (0.44–0.64)	< 0.001	0.38 (0.31–0.47)	< 0.001
Male	0.66 (0.61–0.71)	< 0.001	0.70 (0.65–0.76)	< 0.001
Income categories				
Low	Ref.		Ref.	
Medium	0.82 (0.74–0.91)	< 0.001	0.91 (0.81–1.02)	0.11
High	0.63 (0.54–0.74)	< 0.001	0.81 (0.68–0.97)	0.022
Education level				
Low	Ref.		Ref.	
Medium	1.12 (1.04–1.21)	0.004	1.06 (0.97–1.15)	0.18
High	0.83 (0.76–0.91)	< 0.001	0.87 (0.79–0.97)	0.01
CCI categories				
CCI 0	Ref.		Ref.	
CCI 1	1.19 (1.09–1.31)	< 0.001	1.15 (1.05–1.27)	0.004
CCI > 1	1.77 (1.62–1.92)	< 0.001	1.58 (1.44–1.73)	< 0.001
Psychiatric comorbidity	2.31 (2.07–2.58)	< 0.001	1.50 (1.32–1.70)	< 0.001
Substance abuse	2.85 (2.41–3.36)	< 0.001	1.70 (1.40–2.06)	< 0.001
Opioid use 6 months pre-ICU	7.09 (6.59–7.62)	< 0.001	6.59 (6.11–7.11)	< 0.001
ICU length of stay, days				
0–2	Ref.		Ref.	
3–7	1.28 (1.18–1.38)	< 0.001	1.14 (1.04–1.24)	0.003
> 7	1.14 (0.98–1.31)	0.082	0.82 (0.70–0.97)	0.02
Surgery				
No surgery	Ref.		Ref.	
Elective	0.81 (0.71–0.93)	0.003	1.00 (0.86–1.17)	0.97
Acute care	1.02 (0.85–1.21)	0.87	1.05 (0.86–1.27)	0.65
ICU admission year				
2010–2011	Ref.	Ref.	Ref.	Ref.
2012–2013	0.87 (0.79–0.95)	0.003	0.90 (0.81–1.00)	0.06
2014–2015	0.89 (0.81–0.98)	0.023	0.95 (0.85–1.05)	0.32
2016–2018	0.80 (0.73–0.87)	< 0.001	0.84 (0.77–0.93)	< 0.001

*CCI* Charlson comorbidity index; *ICU* intensive care unit.

During follow-up of 3–6 quarters after admission to critical care, 680 patients passed away, of which 164 were persistent opioid users. In the Cox proportional hazards regression analysis (unadjusted), persistent opioid usage was associated with higher mortality, hazard ratio (HR) of 2.3 (95% CI 2.0–2.8; *P* < 0.001). After adjusting for covariates (age, sex, psychiatric and somatic comorbid conditions, substance dependence and critical care length of stay, the association remained significant, HR of 2.2 (95% CI 1.8–2.6; *P* < 0.001). Also among opioid naïve patients, increased mortality was associated with persistent opioid usage, adjusted HR of 2.3 (95% CI 1.8–2.9; *P* < 0.001).

### Sensitivity analysis

Results did not change when accounting for non-random dropout due to death (data not shown).

### Missing data

Smaller numbers of missing data were found on income (n = 121, 0.4%) and education (n = 514, 1.5%).

## Discussion

In this nationwide cohort study, cardio-thoracic ICU patients exhibited an increased mean opioid consumption prior to ICU admission not returning to baseline levels until 12 months after ICU discharge. Among patients opioid naïve before admission to critical care, mean opioid use remained unchanged in the 8 quarter period follow-up after ICU care. Determinants associated with persistent usage of opioids included younger age, female sex, comorbid conditions, opioid use before admission, and longer stay in the ICU. Mortality 6–18 months after ICU care was higher for individuals with chronic opioid use, including those without prior opioid exposure.

Our study found that cardio-thoracic ICU patients were prescribed more opioids both prior to but also after their critical care episode compared with the general population^20,28,29^. Critical care is complex due to heterogeneity of the patients and the substantial usage before admission might reflect medical conditions and comorbidities associated with increased opioid consumption^30^. Additionally, critical care patients often demonstrate psychiatric comorbidities and substance dependence^31^, both of which have been reported to be associated with chronic opioid use^32^. Mean opioid usage remained elevated for more than a year after admission to critical care compared with baseline use before admission, raising the concern of opioid misuse. A majority of patients do report pain for many years following critical care which is an established risk factor for persistent opioid use^12^.

Many cardio-thoracic ICU patients have undergone elective or acute surgery such as aortic valve surgery or coronary artery bypass graft surgery, and the incidence of severe chronic pain after thoracotomy is up to 50%, suggesting a possible explanation for new persistent opioid use^33^. Another study found that almost 15% of patients with lung cancer continued using opioids three to 6 months post-surgery^34^. Interestingly, among opioid naïve patients, opioid use remained high during the 2-year follow-up, hence the study is not exploring patients already with an ongoing opioid dependence already before critical care admission. In a previous study exploring persistent opioid use in survivors after ICU care, no significant associations were seen between critical care admission and persistent opioid use^35^. Nonetheless, the investigation encompassed subjects from early 2000s and may not accurately represent contemporary prescription tendencies considering the expeditious escalation of misuse within the past ten years.

A number of risk factors for new onset of opioid dependence after cardio-thoracic ICU care were identified, including lower age and female sex. Other studies have reported the findings regarding age but both positive and negative associations for sex^36–38^. New persistent opioid use was also associated with psychiatric disease and this patient group has been shown to have an increased risk of high opioid use also without injury or outside hospital^32^. Opioid use before critical care was associated with persistent opioid use after discharge, a recognized risk factor for persistent opioid usage both among patients warded in the ICU and those undergoing thoracic surgery^9,29,39^. Additionally, patients with new persistent opioid use after critical care had a lower socioeconomic status^40^. Chronic opioid usage was also significantly associated with a higher risk of long-term death after admission to critical care. Several possible mechanisms for increased risk of death among patients using opioids have been explored in previous research, including frequency of stroke^41^, risk of cardiac events^42^, and overdose^43^, as well as delirium, constipation, and respiratory depression^44,45^. Interestingly, in the opioid naïve subpopulation the hazard ratio (HR) was further increased in contrast with other studies in which mortality was increased among persistent opioid users compared with patients not using opioids^46,47^.

Opioids are commonly used in critical care, but long-term use exposes the patients for risk including increased tolerance, dose escalation and hyperalgesia induced by opioids. A majority of survivors after critical care report pain for several years after discharge^48^, and most critical care patients are treated with opioids for sedation or as part of pain management^49^. While it is self-evident that ICU doctors need to treat pain, it is not fully explored for how long opioids should be prescribed. The number of patients using opioids after critical care is not fully understood, nor is the idea that that surgery and critical care might be the starting point of future opioid dependence and misuse. Methods to mitigate the risks associated with opioid administration remain unresolved.

This study comprises all cardio-thoracic ICU cases in Sweden presenting during ten-year period. Strengths include a low rate of loss to follow-up and low rates of missing data. Furthermore, the study is based on high-quality and validated health registers. Limitations include the retrospective and register-based design. Furthermore, we only studied prescribed and dispensed medication, and cannot be sure to what extent the individuals were taking their medication or not. In addition, we have no data on the in-hospital quantities of opioids given to the patients. *In addition, we have no information on type of surgery (cardiac or thoracic). To discriminate between different procedures would be of great interest in future studies.*

## Conclusions

Mean opioid consumption is increased one year after admission to cardio-thoracic ICU care even though no evidence supports long-term use of opioids. For opioid naïve patients, opioid use was increased and without decline two years after ICU care. Younger age, female sex, prior opioid use and comorbid conditions were among the factors associated with new persistent opioid usage both in all patients as well as in the subgroup of opioid naïve patients. Chronic opioid users had an increased risk of death both in the total cohort as well as in the subgroup of opioid naïve patients.

### Supplementary Information

Supplementary Tables.

## Data Availability

The data that support the findings of this study are available from The Swedish Intensive Care Registry and national health registers. Restrictions apply to the availability of these data, which were used under license for the current study, and so are not publicly available. Data is however available from the authors upon reasonable request and with permission of The Swedish Intensive Care Registry and the Swedish National Board of Health and Welfare.
