# Supplementary material for: Opioid use following cardio-thoracic intensive care: risk factors and outcomes: a cohort study
Source: Sci Rep. 2024 Jan 2;14:20. doi: 10.1038/s41598-023-50508-3 (PMC10762227; doi:10.1038/s41598-023-50508-3)
Supplement: Supplementary file 1 — Supplementary Tables. [file 41598_2023_50508_MOESM1_ESM.pdf]

Supplementary Information

TABLE S1 .... **3**
TABLE S2 .... **4**
TABLE S3 .... **5**
TABLE S4 .... **7**
TABLE S5 .... **9**
REFERENCES **11**

Table S1

Included ATC-codes and conversion rates for opioids. Anatomical Therapeutic Chemical (ATC) Classification System codes starting with N02A, Name, Route of administration, Defined Daily Dose (DDD), equianalgesic dose ratios(1-6) and classification as strong or weak opioid.

**ATC**	**Name**	**Route**	**DDD (mg)**	**Equianalgesic dose ratio**	**Strong (S) or weak (W) opioid**
N02AA01	Morphine	PO	100	1	S
N02AA03	Hydromorphone	PO	20	6	S
N02AA05	Oxicodone	PO	75	2	S
N02AA55	Oxicodone combinations	PO	75	2	S
N02AA59 (=N02AJ09)	Codeine combinations excl psycholeptics	PO	100	0.1	W
N02AB01	Ketobemidone	PO	50	1	S
N02AB02	Pethidine		400	0.1	S
N02AB03	Fentanyl	TD	1.2	100	S
N02AC04	Dextropropoxyphene (chloride)	PO	200	0.19	W
N02AC04	Dextropropoxyphene (napsylate)	PO	300	0.19	W
N02AD01	Pentazocin		200	0.17	W
N02AE01	Buprenorphine	TD	1.2	110	S
N02AE01	Buprenorphine	SL	1.2	50	S
N02AG01	Morphine and antispasmodics	PR	30	1.5	S
N02AG02	Ketobemidone and antispasmodics	PR	50	3	S
N02AG04	Hydromorphone and antispasmodics	PR	30	1.5	S
N02AJ06	Codeine and paracetamol	PO	100	0.1	W
N02AJ08	Codeine and ibuprofen	PO	100	0.1	W
N02AJ09	Codeine and other non-opioid analgesics	PO	100	0.1	W
N02AJ13	Tramadol and paracetamol	PO	300	0.2	W
N02AX02	Tramadol	Po	300	0.2	W
N02AX06	Tapendadol	PO	400	0.4	W

PO = per oral, TD = transdermal, SL =sublingual, R = rectal

Table S2

Mean difference in opioid consumption (mg OMEQ) for all consecutive quarters compared to baseline use (equaling the fourth quarter (9-12 months) before ICU admission).

Calendar quarter before/after ICU admission	Mean difference (95% CI), mg OMEQ	P value
-3	5 ((-3)-14)	0.21
-2	5 ((-4)-14)	0.24
-1	12 (0.6-23)	0.040
1 (= first quarter after ICU admission)	285 (269-302)	< 0.001
2	47 (33-62)	< 0.001
3	31 (11-51)	0.002
4	18 (3-33)	0.019
5	11 ((-7)-30)	0.23
6	12 ((-7)-30)	0.22
7	16 ((-8)-40)	0.20
8	14 ((-6)-33)	0.17

OMEQ, Oral Morphine Equivalents.

Table S3

General characteristics for patients admitted to ICU comparing individuals with and without preexisting opioid use 12 months prior to ICU admission.

ICU patients	Preexisting opioid use	No preexisting opioid use
**Count**	4810	29 390
**Age categories**		
**18-45**	247 (5.1)	1829 (6.2)
**46-60**	987 (20.5)	5833 (19.8)
**61-70**	1596 (33.2)	9904 (33.7)
**71-80**	1575 (32.7)	9715 (33.1)
**80-**	405 (8.4)	2109 (7.2)
**Male, count (%)**	3126 (65.0)	21 674 (73.7)
**Income categories, count (%)**		
**Low**	508 (10.6)	2915 (10.0)
**Medium**	3981 (82.8)	23 856 (81.5)
**High**	319 (6.6)	2500 (8.5)
**Education level, count (%)**		
**Low**	610 (33.9)	9193 (31.8)
**Medium**	2133 (45.0)	12 447 (43.0)
**High**	1001 (21.1)	7302 (25.2)
**CCI categories, count (%)**		
**CCI 0**	988 (20.5)	8760 (29.8)
**CCI 1**	1296 (26.9)	9503 (32.3)
**CCI > 1**	2526 (52.5)	11 127 (37.9)
**Psychiatric comorbidity, count (%)**	479 (10.0)	1565 (5.3)
**Substance abuse, count (%)**	191 (4.0)	538 (1.8)
**Acute myocardial infarction**	1535 (31.9)	8634 (29.4)
**Congestive heart failure**	1099 (22.8)	5468 (18.6)
**Peripheral vascular disease**	911 (18.9)	4246 (14.4)
**Cerebrovascular disease**	525 (10.9)	2421 (8.2)

**Dementia**	18 (0.4)	52 (0.2)
**COPD**	815 (16.9)	2924 (9.9)
**Rheumatoid disease**	307 (6.4)	883 (3.0)
**Peptic ulcer disease**	160 (3.3)	444 (1.5)
**Mild liver disease**	119 (2.5)	363 (1.2)
**Moderate/severe liver disease**	27 (0.6)	80 (0.3)
**Diabetes w/o complications**	1215 (25.3)	5971 (20.3)
**Diabetes with complications**	415 (8.6)	1621 (5.5)
**Hemiplegia or paraplegia**	55 (1.1)	180 (0.6)
**Renal disease**	323 (6.7)	1142 (3.9)
**Cancer**	654 (13.6)	2581 (8.8)
**Metastatic cancer**	109 (2.3)	201 (0.7)
**AIDS**	5 (0.1)	33 (0.1)
**ICU length of stay, days**		
**0-2**	3319 (69.0)	21 618 (73.6)
**3-7**	1156 (24.0)	6304 (21.4)
**> 7**	335 (7.0)	1468 (5.0)
**Surgery**		
**Acute care**	368 (7.7)	1833 (6.2)
**Elective**	4108 (85.4)	26 071 (88.7)
**No surgery**	334 (6.9)	1486 (5.1)
**ICU admission year**		
**2010-2011**	1164 (24.2)	6542 (22.3)
**2012-2013**	1018 (21.2)	6415 (21.8)
**2014-2015**	1007 (20.9)	6147 (20.9)
**2016-2018**	1621 (33.7)	10 286 (35.0)

Categorical parameters are presented as n (%), continuous parameters as median with interquartile range (IQR), CCI, Charlson Comorbidity Index; COPD, Chronic Obstructive Pulmonary Disease; AIDS, Acquired Immune Deficiency Syndrome; ICU, Intensive Care Unit.

Table S4

General characteristics in ICU patients stratified by chronic opioid use for a subset of patients not using opioids 12 months prior to ICU admission.

	**No chronic opioid use**	**Chronic opioid use**
**Count**	27 127	2263
**Age categories**		
**18-45**	1635 (6.0)	194 (8.6)
**46-60**	5177 (19.1)	656 (29.0)
**61-70**	9135 (33.7)	769 (34.0)
**71-80**	9151 (33.7)	564 (24.9)
**80-**	2029 (7.5)	80 (3.5)
**Male, count (%)**	20 122 (74.2)	1552 (68.6)
**Income categories, count (%)**		
**Low**	2650 (9.8)	265 (11.8)
**Medium**	22 060 (81.6)	1796 (80.0)
**High**	2317 (8.6)	183 (8.2)
**Education level, count (%)**		
**Low**	8546 (32.0)	647 (29.2)
**Medium**	11 387 (42.6)	1060 (47.9)
**High**	6795 (25.4)	507 (22.9)
**CCI categories, count (%)**		
**CCI 0**	8187 (30.2)	573 (25.3)
**CCI 1**	8816 (32.5)	687 (30.4)
**CCI > 1**	10124 (37.3)	1003 (44.3)
**Psychiatric comorbidity, count (%)**	1352 (5.0)	213 (9.4)
**Substance abuse, count (%)**	445 (1.6)	93 (4.1)
**Acute myocardial infarction**	7901 (29.1)	733 (32.4)
**Congestive heart failure**	5046 (18.6)	422 (18.6)
**Peripheral vascular disease**	3885 (14.3)	361 (16.0)
**Cerebrovascular disease**	2241 (8.3)	180 (8.0)

**Dementia**	49 (0.2)	3 (0.1)
**COPD**	2572 (9.5)	352 (15.6)
**Rheumatoid disease**	813 (3.0)	70 (3.1)
**Peptic ulcer disease**	390 (1.4)	54 (2.4)
**Mild liver disease**	298 (1.1)	65 (2.9)
**Moderate/severe liver disease**	73 (0.3)	7 (0.3)
**Diabetes**	5395 (19.9)	576 (25.5)
**Diabetes + complications**	1446 (5.3)	175 (7.7)
**Hemiplegia or paraplegia**	161 (0.6)	19 (0.8)
**Renal disease**	1057 (3.9)	85 (3.8)
**Cancer**	2343 (8.6)	238 (10.5)
**Metastatic cancer**	177 (0.7)	24 (1.1)
**AIDS**	24 (0.1)	9 (0.4)
**ICU length of stay, days**		
**0-2**	20 044 (73.9)	1574 (69.6)
**3-7**	5739 (21.2)	565 (25.0)
**> 7**	1344 (5.0)	124 (5.5)
**Surgery**		
**Acute care**	1657 (6.1)	176 (7.8)
**Elective**	24 109 (88.9)	1962 (86.7)
**No surgery**	1361 (5.0)	125 (5.5)
**ICU admission year**		
**2010-2011**	5973 (22.0)	569 (25.1)
**2012-2013**	5932 (21.9)	483 (21.3)
**2014-2015**	5670 (20.9)	477 (21.1)
**2016-2018**	9552 (35.2)	734 (32.4)

Categorical parameters are presented as n (%), continuous parameters as median with interquartile range (IQR), CCI, Charlson Comorbidity Index; COPD, Chronic Obstructive Pulmonary Disease; AIDS, Acquired Immune Deficiency Syndrome; ICU, Intensive Care Unit.

Table S5

Univariate and multivariable logistic regression analyses, associations with chronic opioid use for a subset of patients not using opioids 12 months prior to ICU admission presented as OR (95% CI).

	**Univariate**	**P value**	**Multivariable**	**P value**
**Age categories**				
**18-45**	Ref.		Ref.	
**46-60**	1.07 (0.90-1.27)	0.45	1.06 (0.88-1.26)	0.55
**61-70**	0.71 (0.60-0.84)	< 0.001	0.70 (0.59-0.83)	< 0.001
**71-80**	0.52 (0.44-0.62)	< 0.001	0.49 (0.41-0.59)	< 0.001
**80-**	0.33 (0.25-0.43)	< 0.001	0.30 (0.23-0.40)	< 0.001
**Male**	0.76 (0.69-0.83)	< 0.001	0.72 (0.65-0.79)	< 0.001
**Income categories**				
**Low**	Ref.		Ref.	
**Medium**	0.81 (0.71-0.93)	0.003	0.92 (0.80-1.06)	0.25
**High**	0.79 (0.65-0.96)	0.019	0.94 (0.76-1.16)	0.56
**Education level**				
**Low**	Ref.	Ref.	Ref.	
**Medium**	1.23 (1.11-1.36)	< 0.001	1.12 (1.01-1.24)	0.038
**High**	0.99 (0.87-1.11)	0.81	0.92 (0.81-1.05)	0.21
**CCI categories**				
**CCI 0**	Ref.		Ref.	
**CCI 1**	1.11 (0.99-1.25)	0.067	1.13 (1.01-1.27)	0.040
**CCI > 1**	1.41 (1.27-1.57)	< 0.001	1.57 (1.40-1.75)	< 0.001
**Psychiatric comorbidity**	1.98 (1.70-2.30)	< 0.001	1.47 (1.25-1.73)	< 0.001
**Substance abuse**	2.57 (2.05-2.23)	< 0.001	1.77 (1.39-2.26)	< 0.001
**ICU length of stay, days**				
**0-2**	Ref.		Ref.	
**3-7**	1.25 (1.13-1.39)	< 0.001	1.17 (1.06-1.30)	0.003
**> 7**	1.17 (0.97-1.42)	0.098	0.98 (0.80-1.20)	0.84
**Surgery**				

**No surgery**	Ref.		Ref.	
**Elective**	0.89 (0.73-1.07)	0.21	1.00 (0.83-1.22)	0.97
**Acute care**	1.16 (0.91-1.47)	0.24	1.11 (0.86-1.42)	0.42
**ICU admission year**				
**2010-2011**	Ref.	Ref.	Ref.	Ref.
**2012-2013**	0.85 (0.75-0.97)	0.015	0.87 (0.76-0.99)	0.029
**2014-2015**	0.88 (0.78-1.00)	0.055	0.92 (0.81-1.05)	0.22
**2016-2018**	0.81 (0.72-0.90)	< 0.001	0.83 (0.74-0.94)	0.0020

CCI, Charlson Comorbidity Index; ICU, Intensive Care Unit.

References

1. Svendsen K, Borchgrevink P, Fredheim O, Hamunen K, et al: Choosing the unit of measurement counts: the use of oral morphine equivalents in studies of opioid consumption is a useful addition to defined daily doses. *Palliat Med* 2011; 25(7):725-732
2. Mercadante S, Caraceni A: Conversion ratios for opioid switching in the treatment of cancer pain: a systematic review. *Palliat Med* 2011; 25(5):504-515
3. O'Bryant CL, Linnebur SA, Yamashita TE, Kutner JS: Inconsistencies in opioid equianalgesic ratios: clinical and research implications. *J Pain Palliat Care Pharmacother* 2008; 22(4):282-290
4. Sittl R, Likar R, Nautrup BP: Equipotent doses of transdermal fentanyl and transdermal buprenorphine in patients with cancer and noncancer pain: results of a retrospective cohort study. *Clin Ther* 2005; 27(2):225-237
5. Vissers KC, Besse K, Hans G, Devulder J, et al: Opioid rotation in the management of chronic pain: where is the evidence? *Pain Pract* 2010; 10(2):85-93
6. WHO: ATC/DDD Index 2020. Available at: https://www.whocc.no/atc_ddd_index/. Accessed 4 April, 2020
